# New Method of Arresting Gonorrhœa

**Published:** 1880-12

**Authors:** 


### NEW METHOD OF ARRESTING GONORRHŒA.

Dr. W. Watson Cheyne, in a communication to the *British Medical Journal* (vol. ii, 1880, p. 124), expresses the opinion that the extreme contagiousness of gonorrhœa, the existence of a distinct period of incubation, and the steady spread of the inflammation from a given spot, all point strongly to a parasitic origin. Acting on this idea, Dr. Cheyne made a number of cultivation experiments, in which he produced large numbers of organisms in the pus of gonorrhœa. In addition, he believes that the mucous membrane itself in the neighborhood, as well as the pus of the inflamed parts, is the seat of organisms. In the case of gonorrhœa, then, he supposes that at the time of infection a small number of the specific organisms, which in all probability possess a considerable resisting power to the destroying action of the healthy living tissues, are retained in the urethra; that these go on developing; that the products of their growth irritate and weaken the mucous membrane in their vicinity; that the organisms can then penetrate into and live in that weakened tissue; and that the extension of this process over a portion of the mucous membrane of the urethra is the cause of the inflammatory symptoms. Granting the correctness of this theory, what is wanted is a medicinal application which shall destroy the organisms without irritating the inflamed and highly sensitive mucous membrane. Iodoform and oil of eucalyptus appeared to Dr. Cheyne to possess the proper qualities; and he has accordingly experimented with these drugs, mixed with cacao butter, made up into bougies of various lengths. These bougies possess among other advantages over injections this, that, having the diameter of a No. 9 or No. 10 catheter, they expand and smooth out the swollen mucous membrane. Dr. Cheyne has found a combination of the two drugs better than either alone. He employs the following formula: iodoform, five grains; oil of eucalyptus, ten minims, in a bougie of forty grains. The specific element of the disease having been eliminated by this means, antiseptic injections are employed in addition, for the purpose of preventing the discharge from becoming septic and irritating. A saturated solution of boracic acid in water, or an emulsion of eucalyptus oil (one ounce of eucalyptus oil, one ounce of gum acacia, water to forty or twenty ounces), to be used for two or three days. At the end of that time injections of sulphate of zinc, two grains to the ounce, may be begun. The usual precaution of rest, diluent drinks, etc., must be employed. In using the bougies the patient is first told to empty his bladder, partly to clear out his urethra, partly to prevent the necessity of expelling the antiseptic from the canal for several hours. He then lies down on his back, and a bougie from four to six inches long is introduced, and the orifice of the urethra is closed by strapping. The bougie ought to be dipped in eucalyptus oil or in carbolic oil (1-20) before insertion. The patient is instructed to refrain from passing water, if possible, for the next four or five hours. If the case be severe and advanced, he takes another bougie home, and is instructed to introduce it in the same manner after he next passes urine. On that evening, or on the following day, he commences the antiseptic injection, which he uses four or five times daily. On the third or fourth day, when the symptoms have entirely subsided, an injection of sulphate of zinc, two grains to the ounce, is begun. Dr. Cheyne has employed this method in about forty cases, and in all the result has been the arrest of the progress of the gonorrhœa. For a day or two the purulent discharge continues, but afterwards it steadily diminishes in amount, becoming in four or five days mucous, and ceasing altogether in a week or ten days. At the same time the scalding and pain and the symptoms of inflammation rapidly diminish, and disappear completely in about thirty-six to forty-eight hours. In fact, the case becomes no longer one of virulent gonorrhœa, but one of simple urethritis, rapidly progressing towards recovery if properly treated.—*Philadelphia Medical Times.*

